# A Digital Mental Health Intervention for Paranoia (the STOP App): Qualitative Study on User Acceptability

**DOI:** 10.2196/70181

**Published:** 2025-08-07

**Authors:** Laura Eid, Alexandra Kenny, Rayan Taher, Pamela Jacobsen, Che Wei Hsu, Jenny Yiend

**Affiliations:** 1 Department of Psychosis Studies Institute of Psychiatry, Psychology & Neuroscience (IoPPN) King’s College London London United Kingdom; 2 McPin Foundation London United Kingdom; 3 Department of Psychology University of Bath Bath United Kingdom; 4 Social Services (Counselling Specialty) College of Community Development and Personal Wellbeing Otago Polytechnic Dunedin New Zealand; 5 Department of Psychological Medicine Dunedin School of Medicine University of Otago Dunedin New Zealand

**Keywords:** framework analysis, interpretation bias, paranoia, qualitative, digital health technologies, cognitive bias modification

## Abstract

**Background:**

Cognitive bias modification for interpretation (CBM-I) is a technique to modify interpretation and is used to reduce unhelpful negative biases. CBM-I has been extensively studied in anxiety disorders where interpretation bias has been shown to play a causal role in maintaining the condition. Successful Treatment of Paranoia (STOP) is a CBM-I smartphone app targeting interpretation bias in paranoia. It has been developed following research on the feasibility and acceptability of a computerized version. This qualitative study extended that research by investigating the acceptability of STOP in individuals with paranoia. The study design and implementation were informed by the Evidence Standards Framework for Digital Health Technologies (DHTs) published by the UK National Institute for Health and Care Excellence (NICE).

**Objective:**

The aim of the study was to involve service users in the design, development, and testing of STOP and understand the degree of satisfaction with the product. We aimed to establish the extent to which STOP met the NICE minimum and best practice standards for DHTs, specifically its acceptability to intended end users.

**Methods:**

In total, 12 participants experiencing mild to moderate levels of paranoia were recruited to complete 6 weekly sessions of STOP before being invited to a feedback interview to share their experiences. Interview questions revolved around the acceptability of the app, its perceived usefulness, and barriers to the intervention, as well as practicality and views on the use of a digital intervention in principle. Interviews were coded and analyzed using the framework analysis method, combining both deductive and inductive approaches.

**Results:**

Framework analysis yielded 6 themes: independent use and personal fit; digital versus traditional approaches; user reactions and emotional impact; impact on thinking, awareness, and well-being; design, engagement, and usability; and intervention relevance and practical fit.

**Conclusions:**

STOP was found to be a broadly acceptable intervention and was positively received by most participants. The study findings are in line with the NICE Evidence Standards Framework for DHTs, as intended end users were involved in the development, design, and testing of STOP and were mostly satisfied with it. These findings will contribute to the further iterative development of this intervention that targets interpretation bias in paranoia.

**International Registered Report Identifier (IRRID):**

RR2-10.1186/s13063-024-08570-3

## Introduction

### Interpretation Bias and Cognitive Bias Modification

Paranoia refers to unfounded beliefs that others intend to cause harm and exists on a continuum from mild suspiciousness to severe persecutory delusions [1]. Interpretation bias toward threat, defined as the tendency to interpret ambiguous information in a threatening or negative way, is thought to be one of the key cognitive processes involved in the development and maintenance of paranoia. Specifically, individuals with paranoia are more likely to interpret neutral or ambiguous situations as intentionally harmful, often exhibiting hostility interpretation bias (perceiving others as deliberately malicious) and external-personal attribution bias (attributing negative events to the harmful intent of others), as well as biases related to physical threats (such as those involving security devices or medical interventions) [2-6]. While similar biases are seen in anxiety disorders, paranoia is distinguished by its focus on intentional harm, rather than fear of negative evaluation or embarrassment as seen in social anxiety. Cognitive biases, including interpretation bias, have therefore been identified as critical maintenance factors in psychosis and paranoia, highlighting the need for targeted interventions that address these specific processing styles [1,7,8].

Cognitive bias modification for interpretation (CBM-I) is one such technique, designed to reduce interpretation bias toward threat. Research has demonstrated that it is possible to change bias through training [9,10], helping individuals replace maladaptive cognitive patterns with more neutral or positive interpretations. Successful Treatment of Paranoia (STOP) is a smartphone app that delivers CBM-I through a series of ambiguous scenarios, encouraging the user to generate more adaptive interpretations [11]. CBM-I has shown consistent benefits in reducing anxiety and repetitive negative thinking [12-14], but studies applying this approach to paranoia remain limited. One feasibility randomized controlled trial evaluated a desktop version of cognitive bias modification for paranoia (CBM-pa) [15], and a subsequent qualitative study highlighted the need for improvements in the task’s audiovisual design to enhance attention and engagement [16]. These findings directly informed the development of STOP, the smartphone app evaluated in this study.

While STOP draws on CBM principles, it differs significantly from the earlier CBM-pa platform. CBM-pa was delivered in a controlled laboratory setting, whereas STOP is a stand-alone smartphone app designed for independent use. Unlike CBM-pa, STOP enables participants to schedule sessions autonomously, receive automated reminders, and engage with a web-based interface designed to enhance immersion. These structural and experiential differences mean that the findings from the earlier CBM-pa evaluation cannot be assumed to generalize to STOP. A distinct evaluation is therefore warranted.

Thus, a qualitative study, such as the one reported here, is essential to determine whether STOP, in its current form, is a product that would be acceptable to users and suitable for use in routine clinical practice. The importance of gathering this type of evidence for any potential new digital therapy is emphasized in the Evidence Standards Framework for Digital Health Technologies published by the UK National Institute for Health and Care Excellence (NICE) [17], which requires evidence of “acceptability with users” and their involvement in development, design, and testing. The qualitative data were contextualized using the User Experience Questionnaire (UEQ), which quantified participants’ experience of the app, such as their perception of its attractiveness, ease of use, and creativity [18]. Therefore, descriptive statistics of the UEQ are reported to indicate the overall experience of participants using the digital health technology (DHT).

In addition to assessing acceptability, a growing body of literature highlights the importance of applying a human factors lens when evaluating DHTs. This includes attention to usability, accessibility, and alignment with user needs and preferences. Human factors research considers not only whether an intervention works but also how individuals interact with it—factoring in context of use, autonomy, and digital literacy; for example, the concept of the digital therapeutic alliance has gained prominence in recent years, recognizing that users may form relational connections with digital tools, which in turn can affect engagement and perceived effectiveness. Evaluating STOP through both experiential and design lenses enables a more comprehensive understanding of its acceptability and potential for real-world implementation.

### The Rise and Use of DHTs

Digital interventions have become increasingly common over time, and some have proven to be effective [19]. In a scoping review, Zhang et al [19] identified 22 randomized controlled trials exploring the effect of CBM techniques delivered through a web-based intervention and found that CBM could help reduce biases in anxiety disorders and depression in adolescents.

Despite these advancements, there remains a lack of available treatments specifically targeting psychosis and paranoia. Other than pharmacological interventions, the only treatment that NICE recommends for psychosis is cognitive behavioral therapy, a psychological intervention. Smartphone app interventions offer an alternative treatment approach for paranoia. Additional benefits of using smartphone app interventions include their potential to help address long waiting times to access psychological services and to reduce the costs associated with in-person interventions. Furthermore, digital technologies could be considered low-stigma interventions, as service users can access them privately from any location. They could additionally be seen as empowering, enabling service users to take a more active and autonomous role in their care. More specifically for individuals with paranoia, digital health interventions may facilitate help seeking by reducing the need to build a therapeutic alliance and trust in a therapist, both of which can be especially challenging for this population [20,21].

### Aims of This Study

This qualitative study explored users’ views and opinions of STOP, a DHT that targets interpretation bias in individuals with paranoia. The study provided an opportunity for individuals reporting distress from paranoia to voice their attitudes and experiences of using STOP and to offer feedback. The purpose of the study was therefore to explore different views and opinions of the digital intervention and to identify key strengths and weaknesses of the DHT as well as potential improvements. More specifically, the aim was to involve end users in the design, development, and testing of STOP and to assess the extent to which they were satisfied with it. The research questions were therefore formulated as follows:

- To what extent does the smartphone app STOP meet the NICE minimum or best practice standards for DHTs?
- Is STOP acceptable to its intended end users?

## Methods

### Ethical Considerations

This qualitative study received ethics approval from the London-Stanmore Research Ethics Committee (21/LO/0896). Potential participants were emailed a digital copy of the study information sheet and had at least 24 hours to consider participating in the study. All potential participants had the opportunity to ask questions and were free to withdraw at any stage without giving reasons. Interested, eligible participants were then asked to complete an electronic consent. Participants received £40 (US $52) in vouchers as compensation for their time and effort.

### Screening and Recruitment Process

Participants were recruited from various sources, including the South London and Maudsley NHS Foundation Trust’s Psychological Interventions Clinic for Outpatients with Psychosis; Psychological Interventions Clinic for Outpatients with Psychosis clinic at the South West London and St George's Mental Health NHS Trust, through MQ (MQ Foundation, mental health research charity); and the McPin Foundation (a mental health research charity that specializes in integrating lived experiences in research).

Research assistants working on the main STOP randomized clinical trial (ISRCTN17754650) screened participants against the main trial eligibility criteria. Some of the main trial criteria were not relevant for this study, including the following: if on psychotropic medication, no change for at least the last 3 months; interpretation bias screening score of ≥−2; and Positive and Negative Syndrome Scale (PANSS) [22] item 6 score of >3. Therefore, suitable participants who were ineligible for the main trial were redirected to this qualitative substudy and were then assessed against this study’s inclusion and exclusion criteria (Textbox 1 [23]).

**Inclusion and exclusion criteria.**

**Inclusion criteria**

- Self-reported paranoid concerns
- Aged >18 y
- Fluent in written English
- Capacity to consent

**Exclusion criteria**

- Severe cognitive impairment
- Major current physical illness likely to impact participation
- Substance dependence
- Currently receiving another psychological intervention
- Scoring 7 (defined as “extreme”) on the Positive and Negative Syndrome Scale
- At high risk of suicide as per the Columbia Suicide Severity Rating Scale [23]

The choice of the sample size was guided by the concept of information power, which suggests that the number of participants needed for qualitative interview studies decreases as the amount of information held by the sample increases [24]. A smaller sample can be sufficient when the study aim is narrow, the sample is specific, the quality of dialogue is strong, and the analysis is theory informed [24]. Given the focused aim of exploring user experiences of STOP and the relevance of the sample to this aim, the final sample size was deemed appropriate for capturing key insights. Of note, patient and public involvement researcher and coauthor AK also took part in this study as a participant.

### The STOP Intervention

**Figure 1:**
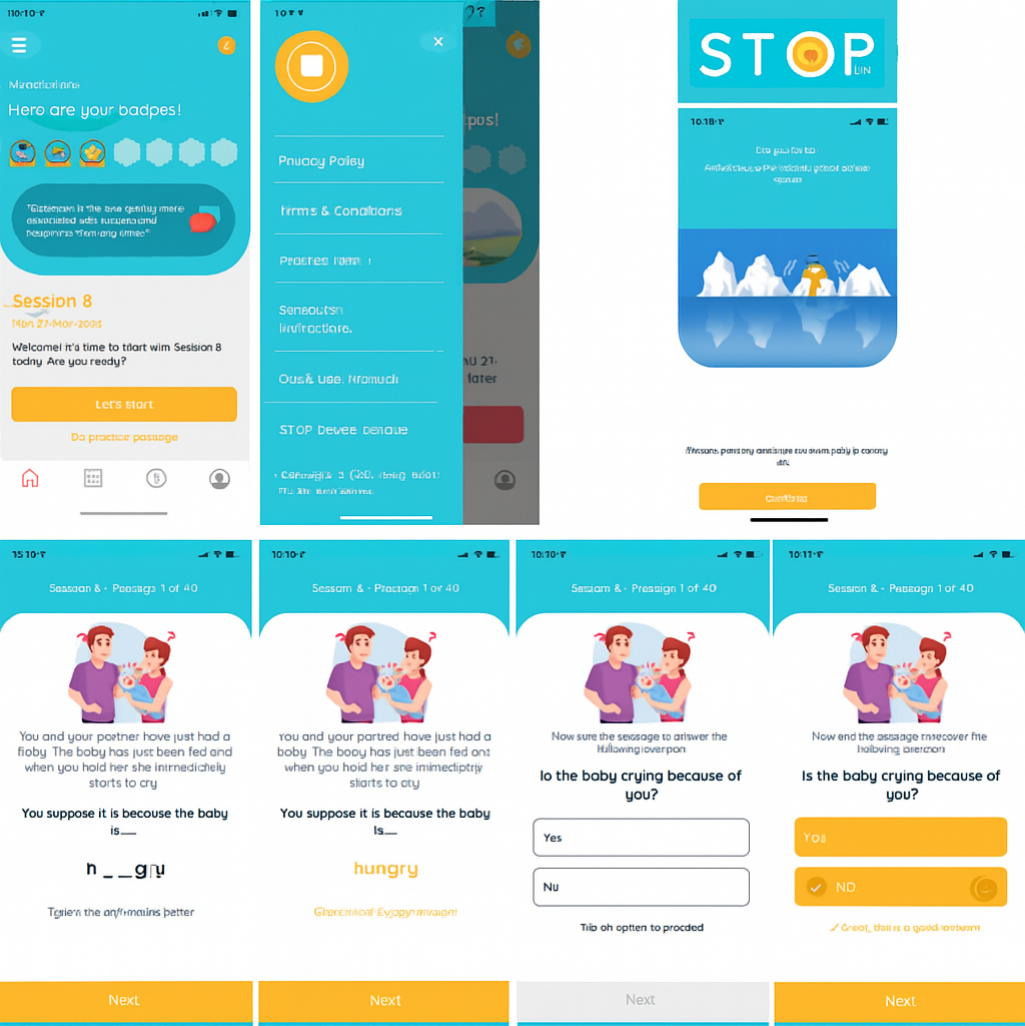
Screenshot of an example of a Successful Treatment of Paranoia intervention item.

STOP was delivered through a smartphone app. It consisted of 40- to 60-minute sessions (depending on reading speed) in which patients were presented with ambiguous scenarios that could be interpreted in either a threatening or a benign way. Each session consisted of 40 scenarios, presented as blocks of 10 items, followed by trivia items to allow users to take a short break (refer to Figure 1 for an intervention item example, a screenshot of STOP’s home page, and a trivia example). After reading each scenario, participants were asked to complete a word with missing letters and a comprehension question, both of which aimed to encourage them to interpret the scenario in a nonthreatening way. Further details regarding the intervention and its development have been published elsewhere [11].

### Materials

The UEQ is a validated and reliable scale designed to measure users’ experiences of using digital products [25]. It consists of 26 items grouped into 6 scales: attractiveness (overall impression; do users like or dislike the product?), perspicuity (is it easy to learn how to use it?), efficiency (can users complete tasks without unnecessary effort?), dependability (does the user feel in control of the interaction with the product?), stimulation (is it exciting and motivating to use?), and novelty (is it innovative and creative? Does it catch users’ interest?). Each item presents a pair of contrasting attributes (eg, annoying-enjoyable and confusing-clear), and respondents are asked to express their agreement with the attributes by selecting the point that most closely reflects their impression of the product. The UEQ demonstrates satisfactory reliability and construct validity [25] and provides normative data from 452 prior digital product evaluations, enabling benchmarking against existing products.

### Procedure

Participants who provided consent were sent a study Android smartphone with the STOP app preinstalled and ready for use. In line with medical device study requirements, hard copy instructions to use the smartphone and the app were mailed to participants’ home addresses. They were then given the opportunity to test the STOP app and were asked to complete 6 weekly sessions of the STOP intervention. They were also offered the option to meet with the researcher (LE) at any time for assistance with navigating the smartphone or app.

All participants had fortnightly check-in calls with the researcher (LE). They were also given the opportunity to ask questions and were offered support for any technical difficulties they encountered.

After the 6 STOP sessions ended, a time was scheduled for a feedback interview and completion of the UEQ. The feedback interviews were semistructured and lasted between 30 and 60 minutes. A topic guide (Multimedia Appendix 1) was used to structure the interview, while allowing flexibility for additional comments. The topic guide was informed by the NICE Evidence Standards Framework for DHTs [17]. Questions revolved around users’ experiences of using the digital intervention, its perceived strengths and weaknesses, their opinions of the visual display of the app as well as the CBM scenarios used, session duration and ease of use, and suggestions for improvement. All interviews were conducted via Microsoft Teams, as all participants had access to a computer and a working internet connection.

After the interviews, participants were asked to complete the UEQ. They could choose to receive a digital version or a printed copy sent to their home address and to complete it either at the beginning of the interview together with the researcher or on their own.

### Data Analysis

UEQ quantitative data were aggregated across participants, and mean scores were plotted against normative data [25] to enable within-sector comparisons.

The qualitative data from the interviews were coded and analyzed using the framework method [26]. The NICE Evidence Standards Framework for DHTs [15] was used to inform the topic guide, codes, and deductive themes. To enhance quality and rigor, the Reflexive Thematic Analysis Reporting Guidelines were followed [27].

Interview recordings were autotranscribed by the software and then manually corrected by LE. Data familiarization was achieved through repeated reading of the transcripts alongside any additional notes made during meetings.

Initial coding was surface level and descriptive and was conducted by LE using NVivo 14 (Lumivero).

A working analytical framework was developed by grouping and categorizing codes. New or revised codes were developed iteratively throughout coding. The data were then summarized in a matrix. Several group discussions between LE, JY, and PJ led to agreement on a final analytical framework and corresponding matrix. Themes were exclusively and intentionally related to users’ experiences of testing, acceptability, and satisfaction with the STOP app. Deductive codes were informed by the topic guide and the NICE Evidence Standards Framework for DHTs [17]. Inductive codes were driven by the data and reflected participants’ unique experiences and language.

### Research Team and Reflexivity

A critical realist postpositivist stance was adopted to account for the fact that the coding and interpretation of data would be impacted by the researchers’ roles in the study. Reflexivity was therefore viewed not as a means of eliminating subjectivity but of making it visible and critically examined throughout the research process. Indeed, based on previous research [28], most researchers involved in the study held beliefs that the app would help reduce interpretation bias in people with paranoia. In addition, the researchers’ own views as well as findings from previous research on individuals’ experiences with smartphone apps and CBM [28,29] would likely influence the analysis and interpretation of data. Using the topic guide informed by the NICE guidelines, interview questions were phrased to avoid guiding or stigmatizing language. The researcher attempted to embrace each participant’s unique and important experiences, as opposed to trying to reach a consensus. A reflective log was maintained throughout recruitment, data collection, and analysis to record reflective thoughts, which were used to inform analysis and interpretation and understand how personal preconceptions and assumptions may influence findings.

## Results

### Overview

We contacted 34 participants to complete 6 sessions of the STOP intervention, followed by a feedback interview. Of these 34 individuals, 16 (47%) met the eligibility criteria and consented to take part in the qualitative study. Of these 16 participants, 4 (25%) dropped out (n=3, 75% reported that they did not feel that they had the time to commit to the study; n=1, 25% was uncontactable), resulting in a final sample of 12 (75%) participants. The participants’ mean age was 36.33 (SD 10.74) years. Of the 12 participants, 7 (58%) identified as female, 8 (67%) were White, and 2 (17%) identified as being from a minority ethnic background (n=2, 17% did not disclose their ethnicity). The mean score on PANSS item 6 was 3.58 (SD 0.79) [22]. Participants’ characteristics and individual scores are summarized in Table 1.

**Table 1:** Participants’ characteristics and User Experience Questionnaire (UEQ) scores.

Participants	Sex	Age (y)	Race and ethnicity	Score on item 6 of PANSS^a^	UEQ attractiveness	UEQ perspicuity	UEQ efficiency	UEQ dependability	UEQ stimulation	UEQ novelty	Sessions completed (n=6), n (%)
1	Male	NR^b^	NR	4	−0.33	−0.25	−0.33	0.25	−0.25	0.00	4 (67)
2	Female	57	White British	3	−1.17	−1.25	−2.00	−0.75	−1.00	−1.00	5 (83)
3	Male	29	Pakistani	3	2.17	2.00	0.75	1.50	1.00	2.25	1 (17)
4	Female	25	Asian	3	1.00	2.00	0.00	0.75	0.75	1.25	3 (50)
5	Male	40	White Irish	4	1.00	1.00	1.50	1.25	0.75	1.25	2 (33)
6	Male	29	White British	5	—^c^	—	—	—	—	—	3 (50)
7	Female	38	NR	4	—	—	—	—	—	—	3 (50)
8	Female	NR	White British	4	—	—	—	—	—	—	3 (50)
9	Female	37	White British	4	1.67	2.00	2.00	1.75	1.75	2.00	5 (83)
10	Female	NR	White British	2	—	—	—	—	—	—	1 (17)
11	Male	47	White British	4	1.33	1.75	2.00	1.50	1.00	0.75	2 (33)
12	Female	25	White British	3	2.67	2.75	1.75	2.00	2.25	1.75	2 (33)

^a^PANSS: Positive and Negative Syndrome Scale (item 6 relates to suspiciousness and persecution).

^b^Not reported.

^c^Missing data.

### UEQ Results

**Figure 2:**
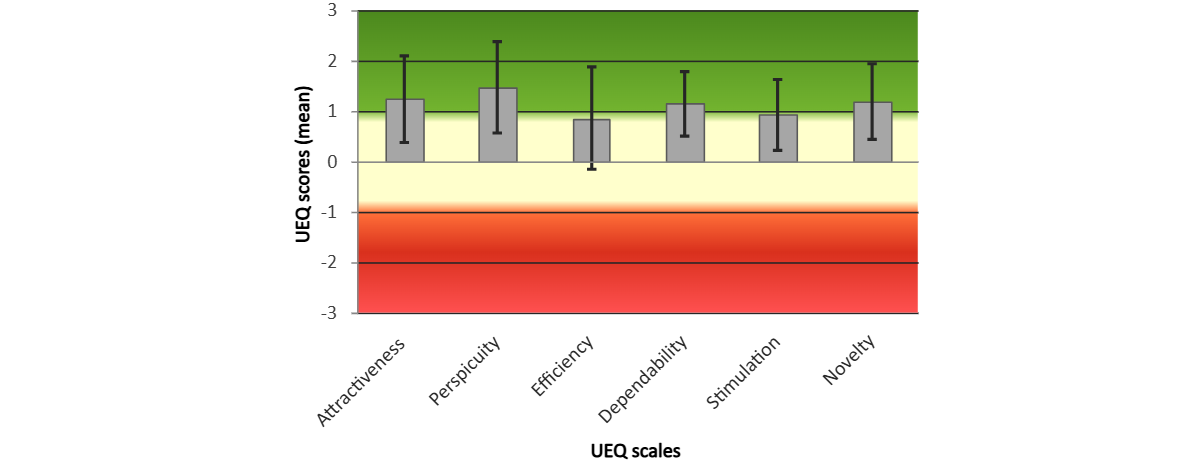
User Experience Questionnaire (UEQ) results. Mean scores on each scale are shown, with error bars representing variance. Green shading indicates a positive evaluation range, cream shading a neutral evaluation range, and red shading a negative evaluation range.

**Figure 3:**
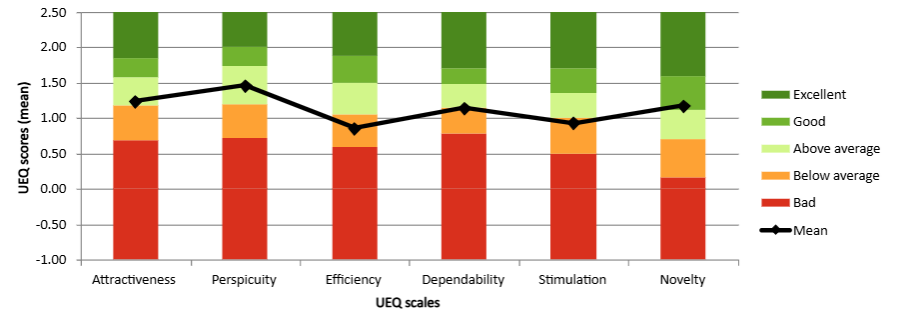
Benchmark graph for Successful Treatment of Paranoia (STOP) based on User Experience Questionnaire (UEQ) scores compared to sector averages. The black data points represent STOP mean scores by scale. The bar colors represent the percentage of other products in the sector falling within that range; for example, a data point falling in pale green (“above average”) indicates that 25% of other products scored higher than STOP, whereas a data point falling in orange (“below average”) indicates that 50% scored higher.

After the feedback interview, 8 (67%) of the 12 participants completed the UEQ. The results are provided in Table 1. UEQ scores range from −3 (maximum negative evaluation) through 0 (neutral) to +3 (positive evaluation). A bandwidth of −0.8 to +0.8 indicates neutral evaluation, with scores outside this range indicating overall negative and positive evaluations, respectively. Participants who completed the UEQ in this study scored an average of between 0.9 and 1.5 on the different scales, which indicates that they described their experience as positive. The UEQ indicated generally positive user perceptions across all subscales, with the highest mean scores observed for perspicuity (mean 1.464, SD 1.302), attractiveness (mean 1.238, SD 1.224), and novelty (mean 1.179, SD 1.087). Lower, yet still positive, ratings were recorded for dependability (mean 1.143, SD 0.923), stimulation (mean 0.929, SD 1.018), and efficiency (mean 0.857, SD 1.457). Participant 1 showed inconsistencies on 5 scales (attractiveness, perspicuity, efficiency, dependability, and stimulation; Table 1, row 1) and was therefore excluded from the reported averages and from the scale mean and variety plots in Figures 2 and 3, as suggested by the UEQ handbook [18]. Specifically, the handbook defines inconsistency as a lack of uniformity in item ratings within a scale. While it may be possible for inconsistencies to occur in a single scale, due to a misunderstanding of the question or an error, it is less likely to be the case when they occur across multiple scales. The handbook therefore suggests excluding such participants from analysis because it is more likely that the behavior indicates random or nonserious completion of the questionnaire.

Summary statistics are presented graphically by scale (Figure 2) and benchmarked against digital sector norms (Figure 3).

### Qualitative Results

#### Overview

Through framework analysis of the interview transcripts, 6 themes were identified, along with topic summaries (summarized in the framework analysis): “independent use and personal fit; digital versus traditional approaches; user reactions and emotional impact; impact on thinking, awareness, and well-being; design, engagement, and usability; and intervention relevance and practical fit.” Table 2 presents the framework analysis, and Figure S1 in Multimedia Appendix 2 illustrates the relationships between themes.

**Table 2:** Framework analysis.

Themes and topic summaries		Example quotations
**Independent use and personal fit**		
	Having agency, taking responsibility, self-discipline, and self-growth	“The advantages are the independence and the self-control and the mindfulness and those skills in themselves, the self-discipline as well, they can [be used] in their own treatments and their own self-discovery journeys.” [Participant 10]
	Matches lifestyle and preferences	“So, like an advantage of this is especially for people who are suffering anxiety and PTSD and don’t get out.” [Participant 5]
	Provides privacy, safety, and flexibility	“When you’re on your own, you sort of have time to think and like go through the answers at your own pace and when it suits you.” [Participant 12]
	Ability, or otherwise, to complete without assistance: neurodiversity, language barrier, and technological proficiency	“I was able to do it myself but for other people, there’s a chance they might need assistance. It could be because they have like dyslexia or there is a language barrier.” [Participant 7]
**Digital versus traditional approaches**		
	Advantages of apps over traditional therapies	“[T]here’s less need for human intervention because the app is friendly and fun.” [Participant 8]
	Disadvantages compared to traditional therapies: digital exclusion, dropout rate, lack of feedback, distractions, and potential dependence on the app	“You don’t get personalized feedback like you would with a person.” [Participant 1]
	Focused, structured, and specific intervention	“[Usually a] therapist can guide it, whereas I like the way that this was more structured and forced you to think through different scenarios. And I like that aspect of it.” [Participant 1]
**User reactions and emotional impact**		
	Perceived as judgmental, patronizing, and invalidating	“There is nothing to validate you or to say your initial response actually had some value in it.” [Participant 1]
	Validating and normalizing	“It is validating. It’s almost as if the researchers have taken into account that we struggle with these thoughts, no matter how insignificant they seem.” [Participant 4]
	Elicited mixed feelings: confusion, excitement, fear and embarrassment, hope, sadness, or neutral	“I think I possibly felt more sad at the end of each session.” [Participant 9] “I would look forward to it, it was interesting.” [Participant 2] “It’s not good, nor bad.” [Participant 7]
	Evocative and triggering of actual experiences	“They’re very good at conveying the paranoid thoughts, they were very evocative and brought up quite a lot of feelings when reading them.” [Participant 9]
	Perceived as restrictive	“One negative thing was that it doesn’t let you say no, I don’t agree with that.” [Participant 5]
**Impact on thinking, awareness, and well-being**		
	Helpful impact on daily life and thinking patterns, sense of achievement, and general positive feelings	“I found it really helpful...it has been giving me some clues like some other way of thinking other than like me, always overthinking what other people’s intentions are.” [Participant 4]
	Offers new perspectives	“I found one of the big values of the app is identifying other options and other ways of looking at things.” [Participant 1]
	Self-reflection, awareness, and understanding of paranoia and its presence in the user’s life	“It has made me realize how insidious paranoia and anxiety are in my everyday life via the scenarios, for which I was always instinctively drawn to the negative assumptions, and so many of which I regularly worry about.” [Participant 9]
**Design, engagement, and usability**		
	Design and look of the app	“I think the color scheme is very calming and soothing...I think it's quite good like, it’s not really intimidating or like causing people distress.” [Participant 4]
	Engagement with the app	“Intuitive, really well designed and written, and very engaging.” [Participant 9] “It was a bit like a reading comprehension for a 7-year-old.” [Participant 10]
	Suggestions for improvement: managing expectations, personalizing the experience, suggestions for design changes, and access to the app	—^a^
**Intervention relevance and practical fit**		
	Acceptability and testing of scenarios	“So, about half I think were quite pretty easy to identify with and about half I had to work a bit harder.” [Participant 1] “I thought they’re all very relatable...there wasn’t a single one that I thought it seemed odd or shouldn’t be in there.” [Participant 8]
	Perceived difficulty of the intervention	“Yeah, I guess the more effort just like you’d have to think about it more to try and work out what that word is instead of like ‘Ohh well, clearly it has this like one word that fits in there.’” [Participant 11] “It was like there’s always a very obvious word that fits in...if it was more challenging, I’d probably focus more on the sentence.” [Participant 12] “I had to think about it more, and that’s not necessarily a bad thing...those were the scenarios which helped me the most.” [Participant 1]
	Session frequency and duration	“I would say it’s just about right, but it’s not a little ask. It took me, I would say just over half an hour...that’s still a fair commitment. Possibly for some people would need it to be a bit shorter.” [Participant 1] “I would like it to be on a continuous basis.” [Participant 4] “It was quite long as well. The sessions were longer than expected...Maybe if the session were half the length as well, then I wouldn’t have got bored so quick so easily.” [Participant 10]
	Views and suggestions related to implementation	“I’m severely disabled, that’s what’s unrelatable. Maybe the app is aimed at people who have less severe disabilities. And yet I did find it helpful.” [Participant 11]

^a^Suggestions are presented in Multimedia Appendix 3.

#### Theme 1: Independent Use and Personal Fit

This theme depicted participants’ attitudes toward completing the intervention on their own and explored the extent to which an autonomous treatment was acceptable. An autonomous treatment was defined here as an intervention to be completed independently, meaning on one’s own and without additional support from professionals or other individuals, aside from any necessary practical or safety checks (eg, in STOP, fortnightly safety check-in calls with the researcher were required). This theme captured users’ views on both obstacles and benefits. Overall, participants seemed satisfied with the intervention being an autonomous treatment.

Some participants indicated that completing STOP on their own gave them a sense of agency and allowed them to take responsibility for their issues and for their progress by deciding to engage with the app and complete sessions on a weekly basis. Some described that STOP helped them monitor their progress and visualize it in the form of badges that they accumulated as they completed sessions. Others thought it could positively impact their self-confidence and allow them to grow:

I would say responsibility because...one of the things that can really happen is that you give up responsibility or you lack confidence, whereas with this it’s like I’m trying, I’m doing it.

Participant 1

Some participants reported satisfaction with the way the intervention was delivered as it fit their preferences and lifestyles. Often, the preferences were associated with mental health conditions getting in the way of seeking help. These participants reported that engaging in a smartphone app was helpful in that it allowed them to access help from their homes. For others, the intervention being an autonomous treatment fit with their personalities and offered them privacy and the space to be honest with themselves as well as the option to complete it at their own pace:

It gives you the independence, the privacy, the self-motivation and discipline.

Participant 10

#### Theme 2: Digital Versus Traditional Approaches

This theme described participants’ acceptability of STOP compared to other interventions, such as cognitive behavioral therapy or counselling. It captured users’ views on both obstacles and benefits related to the intervention’s delivery through a smartphone app. Compared to other treatments, participants seemed to be partly satisfied with the intervention, particularly the fact that it offered flexibility and was specifically focused on paranoia:

This treatment is different because it’s very specific to paranoia and I have not seen any treatment specific to paranoia before.

Participant 8

A few noted disadvantages of smartphone apps in general. These included the need for an internet connection and concerns about digital exclusion. In addition, some of the participants highlighted the high dropout rates associated with app-based interventions, while others reported that it can be distracting, potentially lead to dependence on the app, or not be in line with their preferences. Several comments revolved around the lack of human contact and feedback associated with using an app:

I mean there is an element, I suppose, of the isolation being extended and not having any human support around you, that you would miss out on.

Participant 10

#### Theme 3: User Reactions and Emotional Impact

This theme depicted the extent to which users were generally satisfied with the app after having completed a few sessions. It encompassed a wide range of emotions and reactions that were elicited and labeled. Two contrasting views emerged in the interviews. Most participants described their experiences as positive; however, some expressed negative views, particularly perceiving the app as invalidating or restrictive because it did not allow them the option to disagree with the answers provided within the therapeutic content:

You [are] only allow[ed] to answer the question one way. So not only is it restricting, what are you honestly expecting everyone to have the same opinion?...I can’t give you my opinion if you don’t let me answer the question in a way I feel right in answering it.

Participant 6

I found it validating.

Participant 8

Overall, emotions ranged from confusion and sadness to hopefulness and excitement. Such a range of emotions, even at an individual level, highlighted the unique experience of each participant and the complexities involved. Some participants explored the feelings elicited and used the opportunity to understand themselves better. Others reported that the scenarios presented in the app were evocative because of their relatability. A few described the experience as triggering their paranoia (which is an expected and possible element of STOP’s therapeutic process), whether due to the content of the scenarios or the use of an external smartphone provided by the researcher. However, this was not reported as particularly harmful or distressing. Some participants described STOP as validating because it normalized paranoid thoughts as well as the negative interpretations commonly associated with paranoia.

#### Theme 4: Impact on Thinking, Awareness, and Well-Being

This theme described users’ satisfaction with the app in terms of its impact on their daily lives, changes in thinking patterns, self-reflection, and awareness. Most participants reported that STOP had been helpful in some way.

Some described the impact as emerging from the fact that the app served as a distraction, while others described a sense of achievement from completing the weekly sessions. Some participants highlighted the positive impact on their daily lives, noting changes in their thinking patterns. Some described feeling generally more positive and less stressed after using the app. Some participants, including a few who described the app as restrictive, found it helpful in that it offered alternative ways to interpret situations that they would usually interpret in a threatening or negative manner.

Participants explicitly and spontaneously reported that they perceived the app as a tool for modifying their thinking patterns and also noted that it encouraged reflection and helped them identify themes of paranoia that they related to. They described STOP as helpful in recognizing personal thinking patterns and understanding the extent to which paranoia features in their day-to-day life. Some participants disclosed that the app helped them gain a better understanding of paranoia and how it is perceived by the general population.

#### Theme 5: Design, Engagement, and Usability

This theme outlined participants’ views of the design of the app and explored the extent to which this would be acceptable and satisfying to end users. Participants also spontaneously shared their suggestions for improvement.

Most participants reported feeling satisfied with the design, look, and feel of STOP, including the trivia, audiovisual features, and scheduling and reminder functions. They expressed that they found it “clear” (participant 7) and “not overloading of information” (participant 5). Some reported that they enjoyed the color schemes, the images, and the fact that “it involves all senses” (participant 10), especially if one had “poor eyesight” (participant 1). One participant noted that the images used reinforced binary gender stereotypes, which made the app less relatable. Most users described the app as engaging and interesting, easy to use, and friendly, although a few found it childish. One participant found the app helpful for addressing stigma, noting that its anonymous delivery made it feel discreet. In contrast, another participant felt that the app’s colors and sounds made it stand out and attract unwanted attention (the sounds can be switched off within the app).

Although STOP seemed largely well accepted, participants offered suggestions for improvement. The most common suggestions revolved around offering more response options for the scenarios, personalizing the app, and encouraging more flexible use. Suggestions also included managing expectations and providing more introductory and contextual information about the intervention’s aims, mode of action, and length. Some participants offered alternative design ideas that they believed would make the app more attractive and could help address possible issues of digital exclusion. Additional suggestions included making the app more stimulating and challenging to avoid boredom and to increase engagement:

If it was me, I would leave a section for another option where you could put your own feedback into, like feedback on what you think of the question or if your idea on the scenario didn’t fit into the boxes, boxes of yes or no.

Participant 6

#### Theme 6: Intervention Relevance and Practical Fit

The final theme focused on the core intervention and how it was experienced by users. It reflected the extent to which STOP was perceived as therapeutic, generally acceptable, and satisfying. Some participants indicated that they could strongly relate to the scenarios, while others found some scenarios personally irrelevant. A few reported feeling marginalized or excluded when the scenarios did not reflect the experiences of individuals with disabilities, restrictive lifestyles, or different lifestyle preferences. Some felt that the intervention was too easy and needed to be more stimulating and challenging. They found the more difficult or less relatable scenarios to be the most beneficial. Participants also noted that automatically and systematically completing the missing-letter tasks discouraged them from putting in the effort and therefore did not impact their way of thinking in a positive way.

Most participants commented on the length of the intervention, the frequency of sessions, and the duration of each session. In terms of session duration, most participants felt that the sessions were long and that they would benefit from shorter sessions. Some reported that the session duration interfered with their day-to-day activities and was difficult to fit into their busy schedules, while others said that it suited their lifestyle. Some participants highlighted the benefits of having a weekly session and compared it to therapy, while others expressed a wish to complete the content at their own pace and in shorter blocks throughout the week. In terms of the length of the intervention, some participants reported that 6 sessions would be enough in terms of changing thinking patterns, while others suggested more sessions to enhance the learning. Some participants offered suggestions related to the core intervention, such as shortening the duration of individual sessions, amending the frequency of sessions and length of the intervention, encouraging participants to complete the session, offering the intervention on an indefinite basis, integrating some human contact and additional support if needed, or targeting a specific population.

Other suggestions were related to how participants would envisage the implementation of STOP outside of a research trial:

Well, I guess like if the app was going to sort of develop, maybe integrating some sort of like breathing techniques or something. If things get too much, almost like a little like panic button crisis button on it or something.

Participant 12

I think it should be prescribed by your GP or your psychiatrist or your psychologist or occupational therapist. You should be signposted to it and there’s a mandatory one-to-one onboarding session to answer any questions, to explain and yeah.

Participant 8

The full list of recommendations arising from themes 5 and 6 is provided in Multimedia Appendix 3.

## Discussion

### Principal Findings

This study explored the acceptability of STOP for individuals struggling with paranoia, in the context of the NICE Evidence Standards Framework for DHTs. Overall, participants representing the intended end users expressed broadly positive views of the smartphone app, describing aspects of the experience and design as acceptable and well thought out. These findings are consistent with a previous qualitative study exploring the acceptability of an earlier, computer-based version of the intervention [16]. However, given the modest engagement levels and variation in participant feedback, these results should be interpreted cautiously and viewed as indicative rather than conclusive.

More specifically, after up to 6 weeks of using STOP, participants acknowledged its potential as an autonomous treatment that was not directly supported by a therapist. Most participants reported that the way the intervention was delivered fit their preferences and offered them flexibility and privacy, in line with the perceived advantages of internet-based interventions in previous studies [30,31].

The transdiagnostic approach adopted by STOP avoids the use of stigmatizing diagnostic labels and targets a single symptom by seeking to alter its associated underlying maintaining mechanism.

Our findings suggest that the autonomy of STOP (theme 1) gave users a sense of agency, in contrast to the lack of empowerment frequently experienced by individuals with severe mental health conditions. In this respect, our findings concur with those of others suggesting that DHTs can help empower individuals, offering them more control of their condition and a better understanding of it [32,33].

Of the 12 participants, 1 (8%) reported concerns about data protection, which is common in the context of using DHTs in similar populations [33]. Digital therapies designed for use with those experiencing paranoia will need to pay special attention to transparency and reassurance around data privacy and security if these technologies are to benefit this user group.

Some participants reported that factors related to technical proficiency, neurodiversity, and individual preferences may interfere with users’ ability to benefit from the intervention, highlighting the need to address these considerations in future versions of STOP. While only 1 (8%) of the 12 participants explicitly reported concerns about digital exclusion, this is a commonly reported barrier in the digital mental health literature. By contrast, other circumstances, such as interpersonal challenges, can lead to digital technology improving access to appropriate health care [33,34]. It is likely, then, that some health inequalities may be mitigated by DHTs such as STOP, while others will inevitably be introduced. For this reason, STOP, and DHTs in general, are best seen not as a panacea but as a complement to traditional therapies, with the potential to fill some important gaps in provision.

Some disadvantages of using STOP were also reported (captured in the Theme 2: Digital Versus Traditional Approaches subsection), the most common one being the lack of human contact and feedback—absent from many DHTs and often reported in the literature [32]. Participants offered some suggestions to resolve this perceived disadvantage, such as providing the option to contact a mental health professional as needed while using the app or combining sessions of STOP with traditional psychological therapy. The preference for having alternative options to seek help seems to be common in the context of DHT use [33]. Acceptability was also related to user experience (theme 3). Some participants perceived STOP as judgmental and restrictive, mainly due to the language used within the app (despite it having been codeveloped with users). Others described the intervention as validating and normalizing. Some participants held what seemed to be opposing views of the experience, such as frustration and validation or sadness and hope. This may reflect a common experience of therapy, in which patients may have ambiguous and contradicting feelings toward the approach used, the process of therapy, or their therapist. However, in traditional therapy, the therapeutic alliance is examined, feelings about therapy explored, and approaches adapted to the patients’ needs and feedback. Such a process may not be achievable in digital interventions where the negative aspects of the experience are not addressed in the moment. Future digital therapies may benefit from accommodating this need, for example, by giving participants the opportunity to report their experience in real time. Indeed, the concept of the “digital therapeutic alliance” is gaining traction in psychotherapy literature and provides a suitable theoretical platform from which to explore and improve the digital therapeutic experience [35].

Moreover, some users reported noticing a positive impact of STOP on their lives (theme 4), for example, in terms of their well-being, their thinking patterns, self-reflection, understanding, and awareness of their paranoia. Users also reported general satisfaction with both the core therapeutic intervention and its digital interface (themes 5 and 6). However, some participants expressed that they found a 30- to 40-minute session too long to complete in 1 sitting. These findings echo previous literature on CBM, which has noted that the approach can feel repetitive and monotonous [28,36]. Participants offered some suggestions for improvement, such as shortening the intervention, integrating some additional support, amending the duration of each session, personalizing the scenarios, or making the intervention more challenging and stimulating to optimize changes in thinking patterns.

As shown in Figure 3, STOP received average or above average ratings for attractiveness, perspicuity, dependability, and novelty, but it scored below average on efficiency and stimulation. These findings align with qualitative themes in which participants described the task as repetitive or lacking engagement and occasionally reported that the interface did not facilitate smooth or efficient use. The absence of “good” or “excellent” ratings across scales may reflect the early-stage design of STOP and highlight areas for improvement, particularly in terms of enhancing engagement and interactivity. Given the exploratory nature of this qualitative study and the limited UEQ response rate, we present these results descriptively and cautiously. We have clarified this point in the manuscript and reflected on how this feedback can inform future iterations of STOP.

Finally, it is important to note that engagement with the intervention was low, with only 3 (25%) of the 12 participants completing ≥4 sessions and none completing the full 6-session program. Previous reviews of unguided digital mental health tools have consistently highlighted high dropout rates, with engagement often declining sharply after initial use [37]. In this context, the lack of full intervention completion among our participants aligns with broader trends but still raises important questions about the real-world feasibility of STOP as a stand-alone tool.

Comparisons with similar digital interventions show mixed engagement outcomes; for example, the EMPOWER study reported relatively high engagement when supported by peer involvement [38], while other unguided CBM interventions have faced attrition challenges [28,36]. These findings suggest that embedding some form of human support may be critical for boosting sustained use, particularly in populations with paranoia. Future versions of STOP may therefore benefit from exploring hybrid models that balance autonomy with light-touch support or therapeutic onboarding. Furthermore, the role of the digital navigator is becoming increasingly recognized as a valuable component in the delivery of digital interventions, and this will be an important element to explore in future iterations of STOP [39].

### Strengths and Limitations

A strength of this study is that a critical realist perspective [40] was adopted during the coding and analysis stages, allowing the researchers to reflect on their views and experiences throughout the study and understand how these may have influenced the analytic process. In line with this perspective, a reflective log was maintained throughout the study. To account for the possibility of response and desirability bias during the interviews, the researcher (LE) attempted to normalize all points of view, including negative experiences of using the app. This was achieved by using open-ended questions and encouraging participants to share their own experiences. To avoid steering responses toward the researcher’s expectations, participants were prompted and guided only when necessary. Finally, the researcher adopted a stance of curiosity and nonjudgment during the interviews to encourage participants to share their views openly and without fear of judgment. To our knowledge, this study is the first to explore the acceptability of a digital health intervention delivered via smartphone that targets interpretation bias in individuals with paranoia, in line with the NICE Evidence Standards Framework.

The findings need to be understood in light of possible limitations. Most participants reported mild levels of paranoia, as indicated by their PANSS scores (Table 1). The sample does not therefore reflect the full spectrum of paranoia that will be experienced by the intended end users. In addition, there was an underrepresentation of ethnic diversity in the sample because most of the participants (7/12, 58%) identified as White British. Moreover, none of the participants completed the full 6-week intervention before the interview. One possible barrier may be related to difficulties engaging with apps in psychosis [41], particularly for service users with more severe paranoid symptoms. This partial exposure may have influenced their views on usability, engagement, and perceived benefit. However, even limited engagement was sufficient for participants to form meaningful opinions about key features of the intervention, and themes were largely consistent across those who completed more versus fewer sessions. Low completion rates are not uncommon in digital interventions for psychosis and may reflect broader challenges around attention, motivation, and trust in technology—particularly among individuals with more severe symptoms [41]. Participant feedback in this study pointed to task repetitiveness, lack of stimulation, and external factors (eg, life stressors and environment) as potential barriers. These insights are valuable for informing future iterations of STOP. Future studies could focus on implementing the suggestions offered by participants and reassessing acceptability in line with NICE guidelines. In addition, it could be helpful to explore the acceptability of different intervention doses (eg, 6 wk vs 12 wk), particularly in relation to the efficacy findings reported for each dose in the main trial [42]. It will also be important to understand clinicians’ experiences, opinions, and recommendations regarding STOP, both to improve the intervention further and to assess its “credibility with UK health and social care professionals,” as recommended in the NICE Evidence Standards Framework [17]. One other limitation of this study is that, although participants self-reported experiences of paranoia, their interpretation bias and paranoid symptoms were not formally assessed. While this means that our sample did not correspond to that of the main efficacy trial, it was arguably a more appropriate reflection of the likely population when in clinical use. Finally, the absence of contributors offering external critical perspectives represents a further limitation, which we acknowledge as an area for improvement in future work.

### Conclusions

This study provides evidence of the central involvement of users experiencing paranoia in the qualitative evaluation and further development of the STOP digital mental health intervention. Participants were generally satisfied with STOP, as reflected in the quantitative survey data (UEQ scores) and views expressed during the qualitative interviews. They found the app broadly acceptable and were mostly satisfied with the way it was delivered, the core intervention itself, and the impact it had on their lives. The findings were in line with the target expectations outlined in the NICE Evidence Standards Framework for DHTs [17]. Participants reported that the intervention provided insight and awareness of their paranoid thinking patterns and offered them alternative ways of interpreting situations they related to. Participants also offered some suggestions for improvement that fit with their preferences and condition. This study therefore incorporates suggestions for future development. It will be important to take the findings into account if STOP is to succeed as a new potential intervention targeting interpretation bias toward threat in individuals with paranoia.

## Appendix

Multimedia Appendix 1 Conceptual relationship of themes.

Multimedia Appendix 2 Semistructured interview topic guide.

Multimedia Appendix 3 User interview recommendations for improvement.

## Abbreviations

CBM-I: cognitive bias modification for interpretation
CBM-pa: cognitive bias modification for paranoia
DHT: digital health technology
NICE: National Institute for Health and Care Excellence
PANSS: Positive and Negative Syndrome Scale
STOP: Successful Treatment of Paranoia
UEQ: User Experience Questionnaire

## Disclaimer

For the purposes of open access, the author has applied a Creative Commons attribution (CC BY) license to any accepted author manuscript version arising from this submission.
