# Supplementary material for: A Digital Mental Health Intervention for Paranoia (the STOP App): Qualitative Study on User Acceptability
Source: JMIR Hum Factors. 2025 Aug 7;12:e70181. doi: 10.2196/70181 (PMC12371281; doi:10.2196/70181)
Supplement: Multimedia Appendix 1 [file humanfactors_v12i1e70181_app1.docx]

**Supplementary online material:**

*Supplementary online material 1:* Conceptual relationship of themes


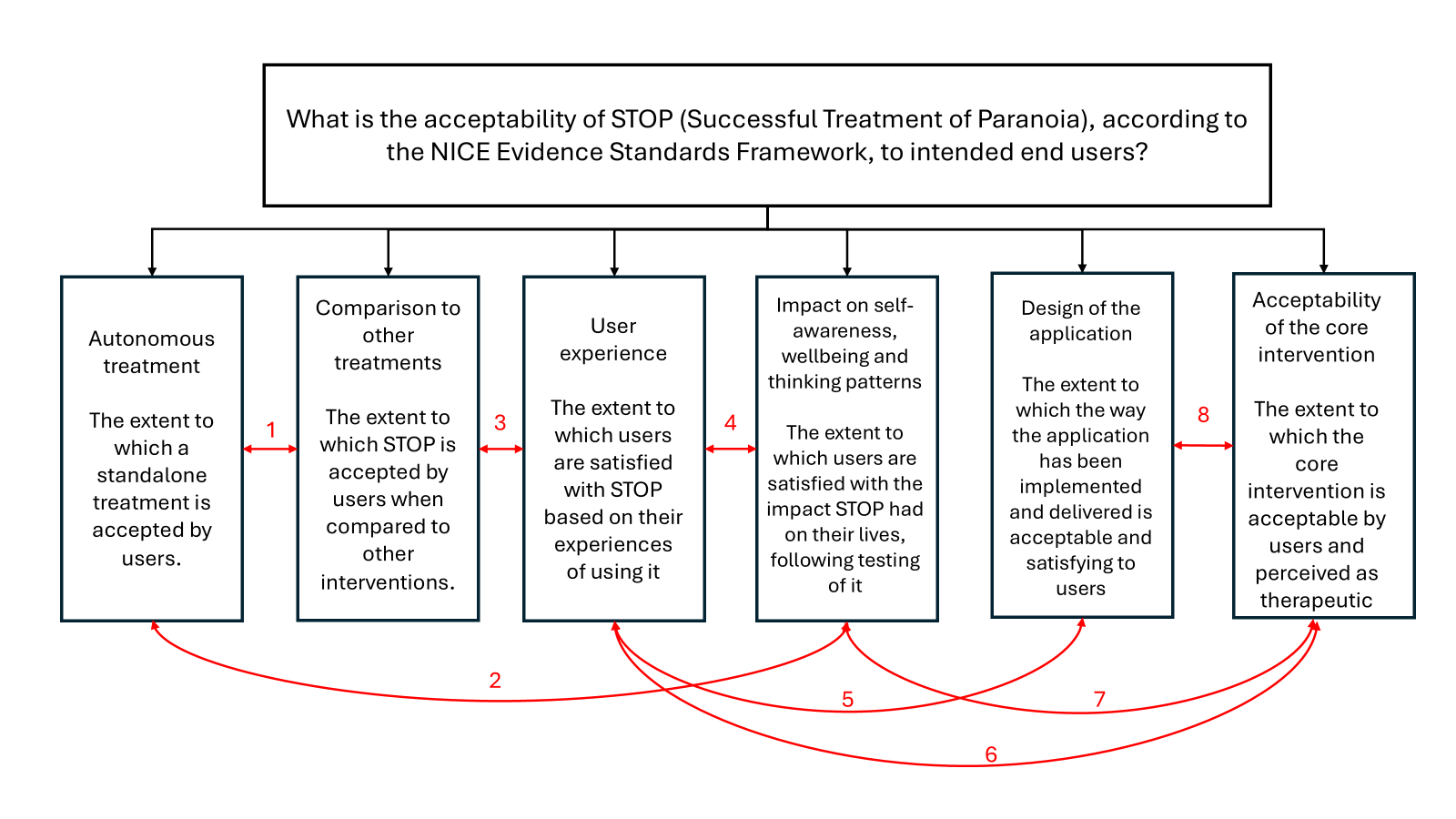


**Figure 4.** Conceptual relationship of themes

The conceptual diagram describes the relationships between the different themes. Each arrow represents a link. 1) The disadvantages of the application, namely a lack of ability to complete the session on one's own due to a lack technological proficiency, language barrier or neurodiversity, influence the degree of acceptability of the intervention as a standalone treatment. Similarly, the advantages of using applications positively influence the acceptability of STOP as a standalone treatment and satisfaction to the user as it matches users’ lifestyles and preferences and provides privacy, safety and flexibility. 2) Taking responsibility to complete the sessions positively impacts thinking patterns and enhances self-growth, confidence and therefore wellbeing. 3) A lack of feedback and human contact can induce feelings of being judged and invalidated. 4) Self-reflection, awareness, and understanding elicit feelings such as sadness and worry but also hope. 5) The design of STOP and user experience are highly interlinked. Feelings elicited and views of the application as restrictive, patronizing or validating are impacted by the way the application has been implemented and delivered. For instance, participants satisfied with the looks and feel of STOP reported excitement. 6) Acceptability of the core intervention and scenarios is associated with feeling validated. For some, the opposite was true where they were less acceptable of the application when they felt judged or restricted in their answers. 7) Completing the application and the core intervention is associated with day-to-day changes as users become aware of alternative interpretations, especially in relatable scenarios. 8) Engagement with the application and views on the perceived difficulty of it are associated. For example, participants who found STOP engaging and stimulating found it more interesting and seem more likely to engage with it, particularly if they were accepting of the duration of the sessions.
