# Supplementary material for: A Digital Mental Health Intervention for Paranoia (the STOP App): Qualitative Study on User Acceptability
Source: JMIR Hum Factors. 2025 Aug 7;12:e70181. doi: 10.2196/70181 (PMC12371281; doi:10.2196/70181)
Supplement: Multimedia Appendix 2 [file humanfactors_v12i1e70181_app2.docx]

**Supplementary online material:**

*Supplementary online material 2: Semi-structured interview topic guide*
