# Supplementary material for: A Digital Mental Health Intervention for Paranoia (the STOP App): Qualitative Study on User Acceptability
Source: JMIR Hum Factors. 2025 Aug 7;12:e70181. doi: 10.2196/70181 (PMC12371281; doi:10.2196/70181)
Supplement: Multimedia Appendix 3 [file humanfactors_v12i1e70181_app3.docx]

**Supplementary material:**

*Supplementary online material 3: User interview recommendations for improvement*

*Headline suggestions:*

1. Response options for the scenarios
   1. Have the option to neither agree nor disagree with the yes or no questions.
   2. Instead of correcting the user, suggest that their answer could be right and gently redirect them to another one. For example, as well as saying, ‘that is one option, what would be another one?’ give some space to add in feedback or another option if they strongly believe it - to make it more validating, less judgmental or restrictive.
2. Personalisation
   1. use scenario content selection to improve relevance to specific user characteristics (family, work, disability, age, gender etc)
   2. Give options to customize e.g. color scheme, rewards, and feedback
3. Flexibility - allow more flexible delivery and usage
   1. completing a session gradually as and when you feel ready instead of in one sitting; give the option for some users to complete each session at their own pace, in shorter blocks, throughout one week.
   2. allow ongoing access to completed sessions.
4. Introduction Manual - provide introductory and contextual information to manage expectations
   1. Explain the intervention’s aims, mode of action and duration
   2. Say what the task consists of, its length, the presence and frequency of breaks

*Additional detailed suggestions*

1. Have a clinician/keyworker present at onboarding and “in the background”.
2. Have the option of reaching out for clinical support –someone to turn to in crisis.
3. Integrate breathing techniques, guided meditation or a panic button.
4. Make it distraction free – suggest users turn notifications off
5. Make the audio more obviously optional – e.g. an in-app button to turn on/off the audio
6. Add encouragements along the way (validate that it can feel long but that you are doing well)
7. Make it less ‘childish’ in terms of task and images (one participant)
8. Tailor the difficulty & demand level to the individual user, for example:
   - Make the tasks increasingly harder, based on the pace at which the answers are given (e.g. the user is quick to answer: make the next item more difficult or vice versa)
   - for those who need it
9. Increase variety to increase stimulation:
   - Make the process of answering more effortful/ varied and less automatic – making it less systematic
   - add variation in how questions are asked (so for example, asking follow-up questions after some scenarios)
10. Permit access to the app through multiple pathways: GP, psychiatrist, other clinicians or openly available to the public
